# Predicting epidermal growth factor receptor mutation status of lung adenocarcinoma based on PET/CT images using deep learning

**DOI:** 10.3389/fonc.2024.1458374

**Published:** 2024-12-13

**Authors:** Lele Huang, Weifang Kong, Yongjun Luo, Hongjun Xie, Jiangyan Liu, Xin Zhang, Guojin Zhang

**Affiliations:** ^1^ Department of Nuclear Medicine, The Second Hospital and Clinical Medical School, Lanzhou University, Lanzhou, China; ^2^ Key Laboratory of Medical Imaging of Gansu Province, Lanzhou, China; ^3^ Gansu International Scientific and Technological Cooperation Base of Medical Imaging Artificial Intelligence, Lanzhou, China; ^4^ Department of Radiology, Sichuan Provincial People’s Hospital, University of Electronic Science and Technology of China, Chengdu, China; ^5^ Department of Nuclear Medicine, Sichuan Provincial People’s Hospital, University of Electronic Science and Technology of China, Chengdu, China; ^6^ Department of Pharmaceuticals Diagnosis, GE Healthcare, Beijing, China

**Keywords:** ^18^F-FDG PET/CT, lung adenocarcinoma, EGFR, mutation, deep learning

## Abstract

**Background:**

The aim of this study is to develop deep learning models based on ^18^F-fluorodeoxyglucose positron emission tomography/computed tomographic (^18^F-FDG PET/CT) images for predicting individual epidermal growth factor receptor (*EGFR*) mutation status in lung adenocarcinoma (LUAD).

**Methods:**

We enrolled 430 patients with non–small-cell lung cancer from two institutions in this study. The advanced Inception V3 model to predict EGFR mutations based on PET/CT images and developed CT, PET, and PET + CT models was used. Additionally, each patient’s clinical characteristics (age, sex, and smoking history) and 18 CT features were recorded and analyzed. Univariate and multivariate regression analyses identified the independent risk factors for EGFR mutations, and a clinical model was established. The performance using the area under the receiver operating characteristic curve (AUC), accuracy, sensitivity, specificity, recall, and F1-value was evaluated. The DeLong test was used to compare the predictive performance across various models.

**Results:**

Among these four models, deep learning models based on CT and PET + CT exhibit the same predictive performance, followed by PET and the clinical model. The AUC values for CT, PET, PET + CT, and clinical models in the training set are 0.933 (95% CI, 0.922–0.943), 0.895 (95% CI, 0.882–0.907), 0.931 (95% CI, 0.921–0.942), and 0.740 (95% CI, 0.685–0.796), respectively; whereas those in the testing set are:0.921 (95% CI, 0.904–0.938), 0.876 (95% CI, 0.855–0.897), 0.921 (95% CI, 0.904–0.937), and 0.721 (95% CI, 0.629–0.814), respectively. The DeLong test results confirm that all deep learning models are superior to clinical one.

**Conclusion:**

The PET/CT images based on trained CNNs effectively predict *EGFR* and non-*EGFR* mutations in LUAD. The deep learning predictive models could guide treatment options.

## 1 Introduction

Lung cancer remains the leading cause of cancer-related mortality worldwide (1, 2). For decades, the standard treatment for advanced non–small-cell lung cancer (NSCLC) relied solely on cytotoxic chemotherapy. However, the introduction of targeted therapy and immunotherapy has rapidly transformed the field of treatment (3). In eligible patients with metastatic NSCLC possessing specific somatic genomic alterations, such as epidermal growth factor receptor (*EGFR*) gene mutations, the response to treatment involves corresponding tyrosine kinase inhibitors (TKIs). TKIs effectively inhibit the activity of abnormal *EGFR* protein kinases, leading to a significant improvement in the objective response rate, extending progression-free survival, and dramatic improvement in quality of life. As a result, they have replaced traditional chemotherapy as a first-line treatment (4–8).

The *EGFR* mutation status is an important predictor of the curative effects of EGFR-TKIs (9, 10). *EGFR* gene status can be categorized into wild-type and mutant types, with mutant types accounting for 40%–50% of the Asian population (11, 12). *EGFR* mutations in exon 19 deletion (*19DEL*) and exon *21 L858R* point mutations are the two most common subtypes in NSCLC, accounting for 90% of all mutations, and referred to as sensitive mutations. Mutations in exons 18 and 20 are relatively rare, and mutations in exon 20 are not suitable for TKIs treatment. Therefore, it is very important to detect the *EGFR* mutation status before treatment. Although molecular pathology is the gold standard, its comprehensive coverage in clinical applications is limited by the heterogeneity of tumors and invasive detection methods (13, 14). Therefore, developing a non-invasive, effective, simple, and practical method for predicting the *EGFR* mutation status is essential, as is screening patients to determine their eligibility for EGFR-TKI treatment.

In recent years, radiomics and deep learning fields have achieved substantial advancements, particularly with the success of deep learning in artificial intelligence due to its powerful feature extraction and classification capabilities, eliminating the need for laborious manual feature extraction.

Notably, deep learning methods have also advanced in research on computed tomography (CT) and positron emission tomography/CT (PET/CT) image prediction of *EGFR* gene mutations. The research team, led by Yunyun Dong, developed a multichannel, multitask, end-to-end deep learning model based on CT images to predict *EGFR* and *KRAS* gene mutations, achieving accuracy rates of 75.06% and 69.64%, respectively (15). Wei Mu et al. constructed a deep learning model that integrated PET/CT images with clinical features to predict *EGFR* mutation status (16). They concluded that the area under the curve (AUC) of the combined model was higher than that of SUVmax, clinical model, and PET/CT deep learning models alone. Guotao Yin and colleagues utilized a squeeze excitation residual network module to construct two deep learning models based on CT and PET images to predict the *EGFR* mutation status. After overlaying the CT and PET images, the AUC value reached 0.84, surpassing the values obtained from using CT or PET alone (17). Although their research has achieved good results, larger sample sizes are still needed to further validate and improve the predictive performance of the model.

Deep learning provides a non-invasive approach for guiding the clinical selection of patients suitable for TKIs treatment. However, due to limited sample datasets, variations in PET/CT examination equipment and differences in convolutional neural networks (CNNs) to extract features, the application of deep learning methods for predicting gene mutations requires additional clinical validation and in-depth research.

Recently, the inception of CNN has shown excellent performance in feature extraction and benign/malignant classification of lung nodules, owing to its multi-scale convolution nuclei and residual structure (18). Additionally, the inception of CNN proves valuable in addressing image dataset problems (19). The Inception V3 architecture, known for superior performance in image detection, classification, and segmentation compared to other deep learning algorithms, has found extensive use in the medical field for classifying and diagnosing various image and video sources, including MRI, CT, microscopy, ultrasound, X-ray, mammography, and color fundus photos (20). However, there is a lack of relevant research reports on whether it can be used to predict EGFR mutations in lung cancer.

This study aims to train and independently validate an *EGFR* mutation state prediction system using PET/CT Inception V3 CNN to screen lung cancer patients for EGFR TKI treatment eligibility. We developed three deep learning models and one clinical model using two retrospective cohorts of patients from two institutions: The Second Hospital of Lanzhou University (LUSH) in Gansu, Lanzhou, China, and Sichuan Provincial People’s Hospital (SPPH) in Sichuan, Chengdu, China.

## 2 Materials and methods

### 2.1 Patient selection

**Figure 1:**
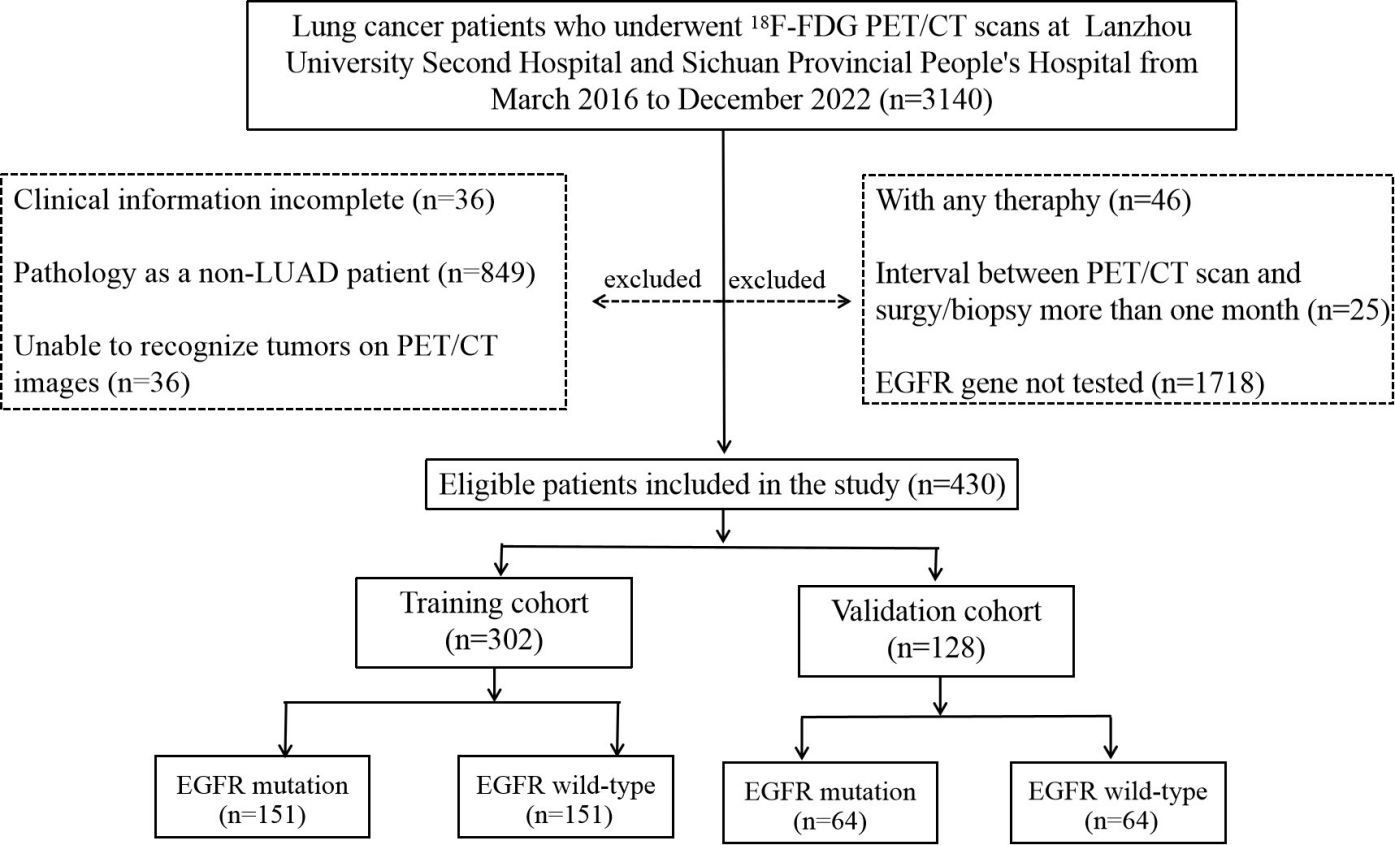
Flow diagram of the recruitment pathway.

This retrospective study was performed in accordance with the ethical standards as laid down in the 1964 Declaration of Helsinki and its later amendments or comparable ethical standards. In addition, this study adhered to the protocol approved by the Institutional Review Board at LUSH and SPPH, with the need for informed patient consent waived. A total of 430 patients who meet the following inclusion criteria were included in two retrospective cohorts accrued from two institutions between March 2016 and December 2022 included in this study. This study was based on the primary tumor of each patient, as some patients have more than one lesion in their lungs. There were 152 patients from LUSH and 278 from SPPH. The cohort coincidentally comprised 215 cases each of wild-type and mutant-type patients. Demographic and clinical information including age, sex, and smoking history was recorded. The criteria used to select patients included: (1) confirmed primary lung adenocarcinoma (LUAD) through a puncture or surgical pathological biopsy; (2) all patients underwent *EGFR* testing, and the results were sensitive mutations; (3) availability of complete PET/CT images from the top of the skull to the upper thigh before puncture or surgery; and (4) comprehensive clinical data. The criteria used for patient exclusion are as follows: (1) confirmation through a puncture or surgical pathology as a non-LUAD diagnosis, such as small-cell carcinoma; (2) patients who underwent radiotherapy, chemotherapy, or targeted drug therapy before PET/CT; (3) the duration between surgery/biopsy and PET/CT imaging exceed 1 month; (4) inability to identify tumors on PET/CT images; and (5) incomplete clinical data. The workflow of this retrospective study is illustrated in **Figure 1**.

### 2.2 *EGFR* mutation analysis

Mutations in *EGFR* exons 18, 19, 20, and 21 were tested with polymerase chain reaction (PCR)–based amplified refractory mutation system by using the human *EGFR* gene mutation detection kit of both institutions (LUSH: Beijing SinoMD Gene Detection Technology Co., Ltd., China; Amoy Diagnostics, Beijing, China; SPPH: Beijing SinoMD Gene Detection Technology Co., Ltd., China; Amoy Diagnostics, Xiamen, China). If they were mutated at any point, then the patient was defined as *EGFR* mutation; otherwise, the patient was considered to having the *EGFR* wild-type.

### 2.3 PET/CT image acquisition

PET/CT scans of LUSH were performed with a Discovery Elite PET/CT scanner (GE Healthcare, WI, USA). Scan parameters were low-dose CT (120 kV, 100 mA), and the slice thickness was 3.75 mm. The initial low-dose CT information was used for attenuation correction. The acquired CT data with a slice thickness of 3.75 mm were reconstructed to 1.25 mm by GE Retro Recon. The reconstruction parameters were as follows: thickness, 1.25 mm; interval, 1.25 mm; Display field of view (DFOV), 70cm; and Recon Type, stand.

PET/CT examination of SPPH was performed with a hybrid scanner (Biograph Duo or Biograph mCTFlow64-4R, Siemens Healthcare Solutions Knoxville, TN). A non-contrast CT scan was firstly performed for localization and attenuation correction, with a slice thickness of 3 mm, interval of 2 mm, a tube voltage of 120 kV, and tube current depending on the patient’s weight. Afterward, PET images were acquired from the base of the skull to the proximal thigh for 3 min per bed position in three-dimensional (3D) mode (Biograph Duo) or continuous table motion acquisitions (Biograph mCT Flow 64-4R). PET images were then reconstructed with an ordered method of ultra HDPET, which included time of flight and resolution recovery (TrueX) information.

### 2.4 CT image interpretation

CT images were interpreted independently by a nuclear medicine physician with 10 years of PET/CT experience and another radiologist with 8 years of CT experience in lung cancer imaging. They were blinded to the clinical data and *EGFR* statuses. All CT images had a thickness of 1.25 mm. A total of 18 CT features were evaluated. The pleural retraction was defined as a pleural movement toward the tumor, whereas an air bronchogram referred to a tube-like or branched air structure within the tumor. Bubble-like lucency denoted a 1- to 3-mm air density area within the mass (21). In regard to the tumor texture, it contains both pure ground glass nodule (GGN) and part-solid GGN. After performing separate evaluations, differences were resolved by consensus.

### 2.5 Tumor segmentation

PET and CT scans were exported in Digital Imaging and Communications in Medicine (DICOM) format at their original resolutions. Two nuclear medicine physicians with experience in PET/CT lung tumor diagnosis manually segmented PET/CT images using ITK-SNAP software (version 3.8.0; www.itksnap.org). Initially, the tumor’s region of interest (ROI) of the tumor was delineated on the CT image, and delineation was completed on transverse lung window (window width, 1,500 HU; window level, −500 HU) and confirmed on coronal and sagittal images. Delineation proceeded from head to toe, encompassing the entire primary tumor and at least 10 mm of its peripheral region was included in the ROI. After each layer, was delineated, a 3D volume of the region of interest (VOI) was generated. For PET segmentation, the tumor’s location on CT is first located to avoid misidentification when the tumor metabolism is low and semi-automatically drawn by referencing to the ROIs with a standard uptake value threshold of 40%. The two reviewers were unaware of the patient’s clinical data and *EGFR* test results while outlining tumor VOI. The Kappa test assessed the consistency of VOI-extracted features between the two reviewers using intra/inter-class correlation coefficients (ICCs) as evaluation indicators. The closer the ICC value is to 1, the higher the consistency, and vice versa.

### 2.6 Data pre-processing

In the context of image preprocessing for CT data, the original images are first adjusted to a window width and level setting of −500 and 1,500, respectively. Following this adjustment, the data undergo a 1 × 1 × 1 resampling process. Subsequently, the CT images are cropped on the basis of the maximal edge range of the 3D ROI. For each case, eight central images from the lesion area are retained as input data (the eight central images of the lesion refer to the eight-layer coronal images of the central slice of the 3D lesion). Similarly, in the case of PET images, after completing the SUV conversion using LIFEx software (version v6.20) (22), the images are cropped on the basis of the maximal edge of the 3D ROI. Again, eight central images from the lesion are preserved as input data for each case. If the number of lesion slices is less than eight, then all slices of data are kept as input. The images are then resized to 299 × 299 pixels to satisfy the input requirements of the CNN model. The data are randomly stratified into training and validation sets in a 7:3 ratio.

### 2.7 Deep learning model training and validation

This network was obtained from an open-access library (Keras Applications, https://keras.io/applications/). The deep learning network platform was Python keras. PET/CT images were used to construct three models: CT, PET, and CT combined with PET (PET + CT). All three models underwent training using Keras version 2.3.1 with TensorFlow 2.3.0 as the supporting backend.

Initially, the network’s weights were set using the pretrained weights from the ImageNet model. Subsequently, a Global Average Pooling 2D layer was added, averaging the pooling across each feature map to transform it into a fixed-size vector for processing by subsequent fully connected layers. Following this, a fully connected layer with 256 neurons was incorporated, utilizing the rectified linear unit (ReLU) activation function to introduce non-linearity. A dropout layer was then added after this layer to mitigate the risk of overfitting. The final step involved adding an output layer with a softmax activation function, designed for multiclass classification. The loss function chosen was “binary_crossentropy,” which is appropriate for binary classification tasks. For optimization, the Adam optimizer was selected with a learning rate set at 0.0001, regulating the speed of weight updates.

**Figure 2:**
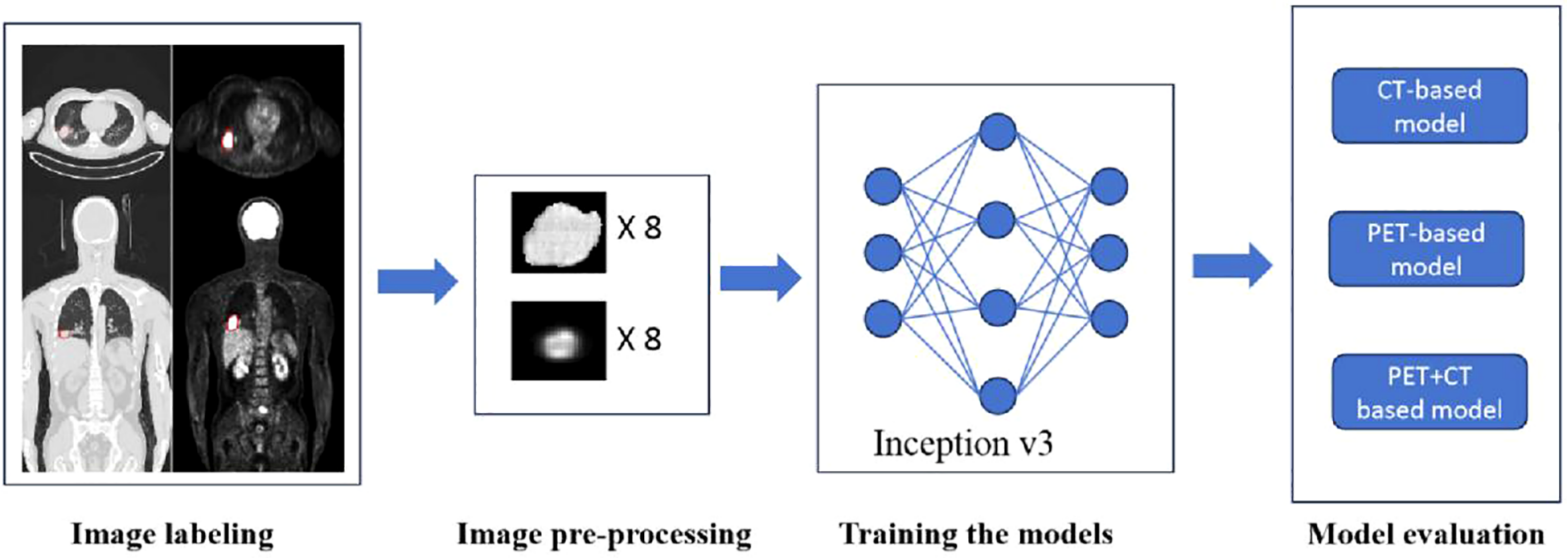
Process diagram for training and testing deep learning models.

Process diagram for training and testing deep learning models are shown in **Figure 2**. The models were trained with separate inputs of CT data, PET data, and combined PET + CT data, resulting in the development of three deep learning models based on the Inception V3 architecture.

### 2.8 Model interpretation

In order to visualize the prediction of *EGFR* mutation status by deep learning models, we applied Gradient weighted Class Activation Mapping (Grad-CAM) technology (23), which can help us understand how the model classifies based on the features of the input image. We chose the last convolutional layer of the network because it contains both advanced features and spatial information. Then, we propagated the image forward to obtain a score for a specific category, calculated this score relative to the gradient output of the selected convolutional layer, weighted each channel using the global average of these gradients, and finally obtained a weighted heatmap with the same spatial dimension as the convolutional layer.

### 2.9 Statistical analysis

All data were statistically analyzed using R software (version 4.1.3). The significance level used throughout this study was 0.05. For clinical and imaging features, univariate and multivariate regression analyses were used to screen for independent risk factors associated with *EGFR* mutations, and a clinical model was established. Student’s t-tests or Mann–Whitney U tests were used for continuous variables, and chi-square or Fisher’s exact tests were employed for categorical variables. Receiver operating characteristic (ROC) curve analysis, along with the AUC value, accuracy, sensitivity, specificity, recall, and F1 values, was used to assess the predictive performance of various models. The DeLong test was used to compare the differences in ROC curves between the various models.

## 3 Results

### 3.1 Clinical characteristics

This study included 430 patients with NSCLC in the final analysis, comprising 229 men (53.3%) and 201 women (46.7%), with an average age of 60.56 ± 10.72 years, their ages varied from 27 to 85 years. The cohort consisted of 215 wild-type and 215 *EGFR* mutations. We employed stratified sampling, dividing to the study cohort into training (n = 302) and validation (n = 128) cohorts in a 7:3 ratio for model building and validation, respectively. **Table 1** presents the clinical characteristics of the training and testing datasets.

**Table 1:** Patient information in the training dataset and the testing dataset.

Characteristics	Training dataset (N = 302)	Testing dataset (N = 128)	*P-*value
Gender(%)			0.972
Female	141 (46.7%)	60 (46.9%)	
Male	161 (53.3%)	68 (53.1%)	
Age (years)	60.39 ± 10.74	60.96 ± 10.70	0.614
Range	27–84	34–85	
Smoking history			0.737
Smoker	97 (32.1%)	39 (30.5%)	
Non-smoker	205 (67.9%)	89 (69.5%)	
EGFR			1.000
Mutant	151 (50.0%)	64 (50.0%)	
Wild type	151 (50.0%)	64 (50.0%)	

### 3.2 Predictive performance of different models

**Figure 3:**
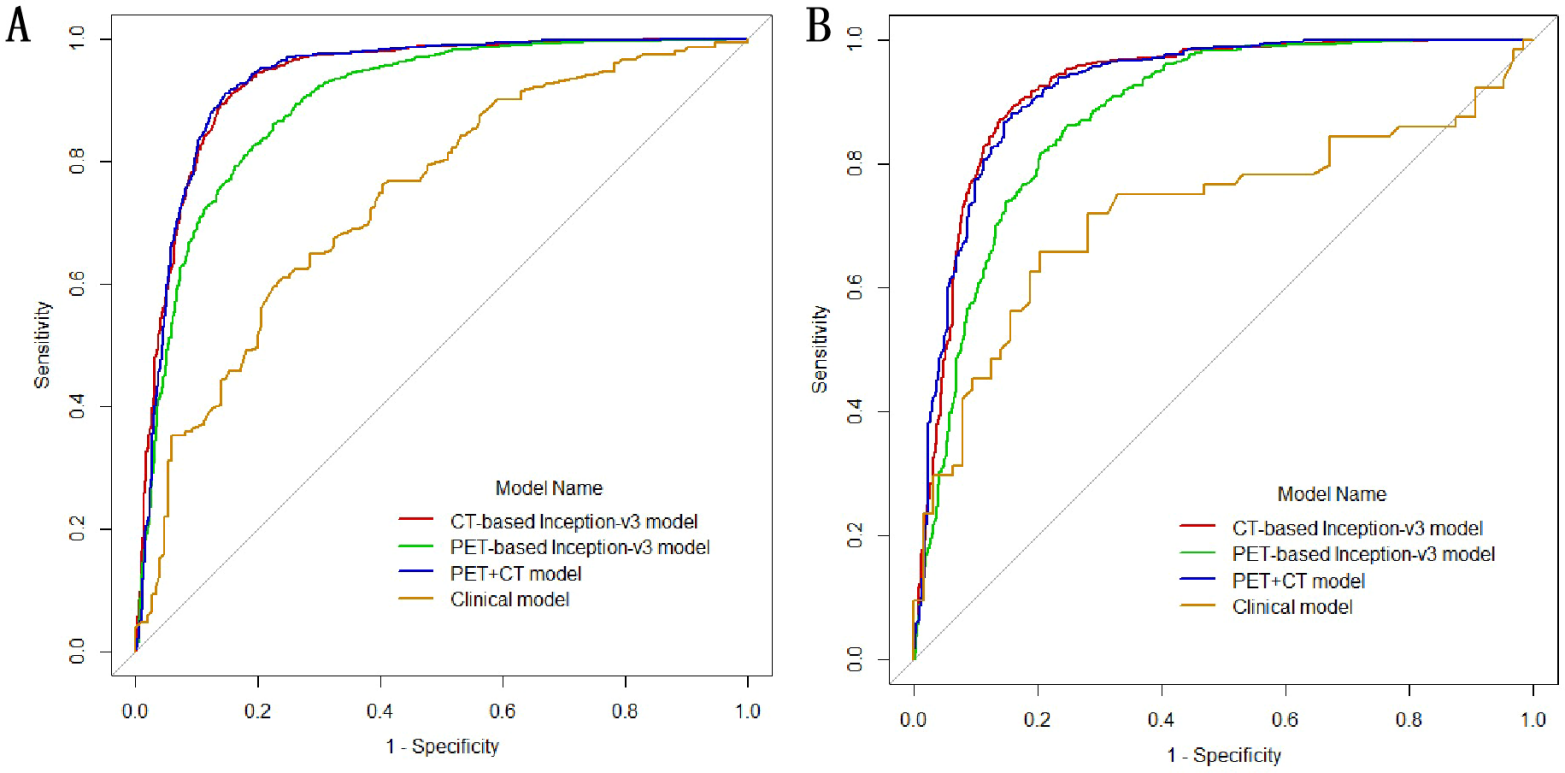
Receiver operating characteristic (ROC) curves of the different models in training **(A)** and validation cohorts **(B)**.

In the univariate logistic regression analysis, sex, age, smoking history, margin definition, longest diameter, short diameter, speculation, pleural retraction, bubble-like lucency, air bronchogram, vascular convergence, texture, calcification, and lymphadenopathy (all P < 0.05) were associated with *EGFR* mutations. In the multivariate logistic regression analysis, the longest diameter, pleural retraction, air bronchogram, and calcification were the independent predictors of *EGFR* mutations (**Table 2**). The clinical model formula is 0.182–0.027 × longest diameter + 0.885 ×pleural retraction + 1.52 × air bronchogram + 1 × calcification. The AUC, accuracy, sensitivity, specificity, recall, and F1 values of the different models on the training and validation sets are listed in **Table 3**. Among the four models, the deep learning model based on CT images had the highest predictive performance, followed by the PET + CT, PET, and clinical models. In the training set, the AUC for CT, PET, PET + CT, and clinical models were 0.933 (95% CI, 0.922–0.943), 0.895 (95% CI, 0.882–0.907), 0.931 (95% CI, 0.921–0.942), and 0.740 (95% CI, 0.685–0.796), respectively, whereas, in the testing set, they were 0.921 (95% CI, 0.904–0.938), 0.876 (95% CI, 0.855–0.897), 0.921 (95% CI, 0.904–0.937), and 0.721 (95% CI, 0.629–0.814), respectively. ROC curves of the different models in training and validation cohorts are shown in **Figure 3**.

**Table 2:** The relationship between clinical variables of patients and EGFR mutation status (wild-type vs. mutation) in the training set.

Characteristics	EGFR wild-type N = 151	EGFR mutant N = 151	Univariate analysis		Multivariate analysis	
			OR (95% CI)	*P*-value	OR (95% CI)	*P*-value
Gender, %			0.26 (0.16,0.42)	<0.001		
Male	105 (69.5)	56 (37.1)				
Female	46 (30.5)	95 (62.9)				
Age (years)	63.0 [54.5; 70.0]	58.0 [52.0; 66.0]	0.97 (0.95, 0.99)	0.007		
Smoking history, %			0.56 (0.34, 0.91)	0.020		
Yes	58 (38.4)	39 (25.8)				
No	93 (61.6)	112 (74.2)				
Distribution, %			0.81 (0.39, 1.68)	0.580		
Central	15 (9.93)	18 (11.9)				
Peripheral	136 (90.1)	133 (88.1%)				
Lobe location, %			0.91(0.78,1.05)	0.192		
Right upper	46 (30.5)	56 (37.1)				
Right middle	3 (1.99)	7 (4.64)				
Right lower	33 (21.9)	25 (16.6)				
Left upper	38 (25.2)	37 (24.5)				
Left lower	31(20.5)	26(17.2)				
Shape, %			1.18 (0.74, 1.87)	0.481		
Regular	94 (62.3)	88 (58.3)				
Irregular	57 (37.7)	63 (41.7)				
Margin, %			0.47 (0.23, 0.97)	0.040		
Well-defined	126 (83.4)	138 (91.4)				
Poor-defined	25 (16.6)	13 (8.61)				
Longest diameter	34.0 [25.5; 47.5]	31.0 [24.5; 39.0]	0.98 (0.97, 1)	0.011	0.97 (0.96, 0.99)	<0.001
Short diameter	25.0 [17.0; 33.5]	23.0 [17.5; 28.5]	0.98 (0.96, 1)	0.018		
Spiculation, %			2.3(1.45,3.65)	<0.001		
Yes	63 (41.7)	94 (62.3)				
No	88 (58.3)	57 (37.7)				
Lobulation, %			1.08 (0.63, 1.85)	0.783		
Yes	115 (76.2)	119 (78.8)				
No	36 (23.8)	32 (21.2)				
Pleural retraction, %			2.92(1.81, 4.72)	<0.001	2.42 (1.46, 4.02)	<0.001
Yes	42 (27.8)	80 (53.0)				
No	109 (72.2)	71 (47.0)				
Bubble-like lucency, %			2.01 (1.17, 3.46)	0.011		
Yes	27 (17.9)	46 (30.5)				
No	124 (82.1)	105 (69.5)				
Air bronchogram, %			3.79 (2.06, 6.96)	<0.001	4.57 (2.31, 9.06)	<0.001
Yes	17 (11.3)	49 (32.5)				
No	134 (88.7)	102 (67.5)				
Vascular convergence, %			1.75 (1.1, 2.78)	0.019		
Yes	81 (53.6)	101 (66.9)				
No	70 (46.4)	50 (33.1)				
Texture, %			2.99 (1.47, 6.08)	0.002		
Solid	139 (92.1)	120 (79.5)				
Non-solid	12 (7.95)	31 (20.5)				
Necrosis, %			0.76(0.41,1.38)	0.361		
Yes	29 (19.2)	23 (15.2)				
No	122 (80.8)	128 (84.8)				
Cavitation, %			1.37 (0.63, 3.01)	0.429		
Yes	12 (7.9)	16 (10.6)				
No	139 (92.1)	135 (89.4)				
Calcification, %			2.19 (1.05, 4.56)	0.036	2.72 (1.23, 6)	0.011
Yes	12 (7.95)	24 (15.9)				
No	139 (92.1)	127 (84.1)				
Pleural effusion, %			1.23 (0.7, 2.16)	0.474		
Yes	28 (18.5)	33 (21.9)				
No	123 (81.5)	118 (78.1)				
Lymphadenopath, %			0.54 (0.34, 0.86)	0.010		
Yes	71 (47.0)	49 (32.5)				
No	80 (53.0)	102 (67.5)				

CI, confidence interval; EGFR, epidermal growth factor receptor; OR, odds ratio; SD, standard deviation; vs., versus.

**Table 3:** Predictive performance of different models in the training and testing datasets.

Modality	AUC	Accuracy	Sensitivity	Specificity	Recall	F1-value
Training set						
CT	0.933 (0.922–0.943)	0.874	0.911	0.844	0.911	0.869
PET	0.895 (0.882–0.907)	0.815	0.862	0.775	0.862	0.811
PET + CT	0.931 (0.921–0.942)	0.880	0.908	0.856	0.908	0.873
Clinical	0.740 (0.685–0.796)	0.685	0.609	0.762	0.609	0.659
Testing set						
CT	0.921 (0.904–0.938)	0.959	0.908	0.814	0.908	0.856
PET	0.876 (0.855–0.897)	0.803	0.862	0.754	0.862	0.800
PET + CT	0.921 (0.904–0.937)	0.852	0.894	0.814	0.894	0.850
Clinical	0.721 (0.629–0.814)	0.719	0.625	0.812	0.625	0.690

### 3.3 Comparison of the efficiency of different models

Efficiency comparison of different models using the DeLong test revealed significant statistical differences in the performance of different models. The results indicated that the CT-based deep learning model had notable statistical differences compared to the PET model and clinical models in the training set. However, there was no observed statistical difference in performance compared to the PET + CT model. A significant statistical difference was present between the PET, PET + CT, and clinical models. Similar statistical results were obtained in the test sets, as shown in **Table 4**.

**Table 4:** DeLong test for the predictive performance of different models in the training and testing sets.

Models	CT deep learning model	PET deep learning model	PET + CT deep learning model	Clinical model
CT deep learning model	/	0.001	1	<0.001
PET deep learning model	**<0.001**	/	<0.001	
PET + CT deep learning model	**0.9**	**<0.001**	/	<0.001
Clinical model	**<0.001**	**<0.001**	**<0.001**	/

Bold font represents the results of the training set, whereas normal font represents the results of the testing set.

### 3.4 Model visualization

**Figure 4:**
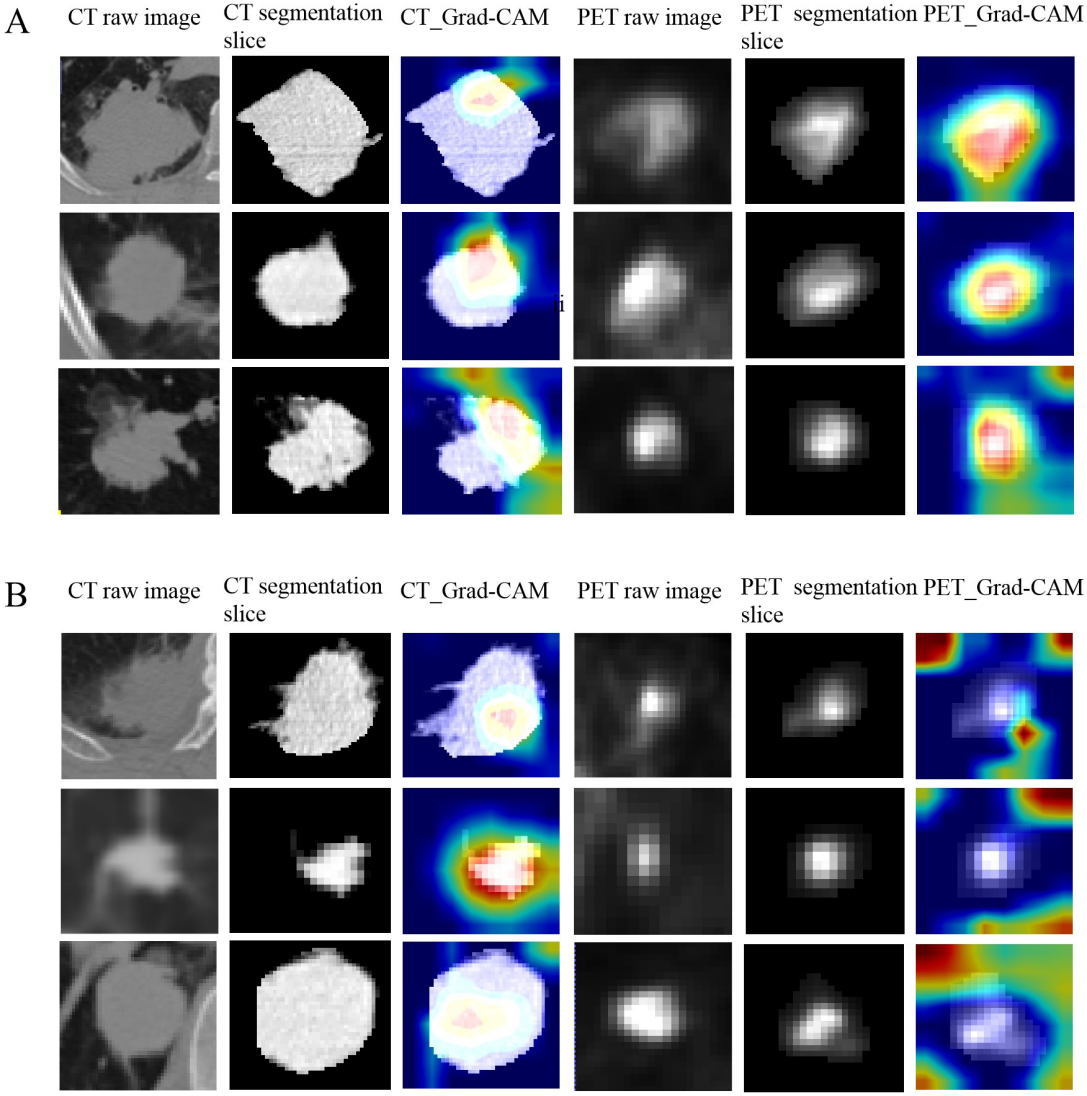
The predictive process of deep learning models using Grad-CAM algorithm. **(A**, **B)** show examples of three representative patients with EGFR mutant and wild-type, respectively. The first and forth columns are CT and PET raw image of tumor. The second and fifth columns are CT and PET central segmentation slices of the tumor, respectively. The third and sixth columns are the CAM (class activation map) images of the CT and PET central segmentation sections of the lesion, respectively. **(A)** The results showed that CT models tended to highlight the areas near the edge of the tumor and PET models frequently annotated a more diffuse region around the center of the lesion as EGFR mutant type. **(B)** CT models based on the pattern of central areas and PET models based on the pattern of the surrounding area of the tumor to explain these tumors as wild-type ones.

**Figure 4** showed the predictive process of our deep learning model using Grad-CAM algorithm. In **Figure 4A**, the results showed that CT models tended to highlight the areas near the edge of the tumor and PET models frequently annotated a more diffuse region around the center of the lesion as *EGFR* mutant type, whereas in **Figure 4B**, CT models based on the pattern of central areas and PET models based on the pattern of the surrounding area of the tumor explain these tumors as wild-type ones.

## 4 Discussion

In this study, we employed traditional methods to construct clinical models and utilized the Inception V3 deep learning framework to build multiple models of CT, PET, and PET + CT for predicting *EGFR* mutation status in LUAD. Our study revealed that CNNs trained with FDG-PET/CT data performed well in predicting *EGFR* mutation status in lung cancer. The results indicated that the deep learning model based on CT images outperformed PET-only and clinical-only models significantly and performed similarly to PET + CT. CT and PET predictive performances showed statistical differences compared to clinical model, highlighting the efficacy that deep learning methods are superior to traditional clinical predicting *EGFR*. The AUC and accuracy of CT-based models exceed 90%, whereas PET-based models achieve an AUC of over 85% and an accuracy of over 80%. Our model’s superior predictive performance surpasses that of some previous studies (17, 24); this can be attributed to the following reasons. Firstly, we chose the advanced network architecture Inception V3 for medical image classification. ResNet, VGG, DenseNet, and Inception V3 are well-known CNN architectures in the field of deep learning, each with unique design concepts and application scenarios. VGG is known for its simplicity and depth, ResNet solves the problem of deep network training through residual connections, DenseNet improves parameter efficiency and gradient propagation through dense connections, and Inception V3 improves performance by capturing information at multiple scales. Each architecture has its own advantages and applicable scenarios. There are studies indicating that Inception V3 has high accuracy and practicality in medical image classification tasks, and our research also belongs to the field of medical image classification.

Currently, in the field of medical image analysis, CNNs are recognized as the most prominent deep learning architectures (25). A CNN comprises of using three key components: an input layer, multiple, hidden layers, and an output layer. The hidden layers typically include convolutional, pooling, and fully connected layers, which collectively process the data.

Inception V3 belongs to the GoogLeNet family, which is based on the idea that most activations in deep CNNs are either unnecessary or redundant because of the high correlations between them. Therefore, reducing the connections between network layers, resulting in sparse weight/activation, makes it more efficient. GoogLeNet thus proposed a module called “inception” that approximates a sparse CNN with a normal dense construction. As the GoogLeNet family evolves, additional well-tuned features are incorporated into the network such as stacked 3 × 3 kernels (Inception V2), the Batch Normalization layer (Inception V3), and a combination of “residual” and “Inception” modules (Inception-ResNet V2).

Another reason why our deep learning model has high predictive performance may be that we used manual tumor segmentation. Research reports have shown that the use of manual segmentation can improve diagnostic accuracy (26).

Previous deep learning studies suggest that combining CT and PET images can enhance the model’s predictive performance, indicating that the PET model outperforms the CT model in predictive performance (17, 24). However, our results contradict this trend. This could be attributed to the following reasons: Firstly, unlike simple and traditional architecture, deep CNN algorithms efficiently perform edge detection through multiple convolutional and hidden layers with hierarchical feature representations, favoring CT images (27). Second, we found that the main reason for false positives and false negatives in our models is the reconstruction algorithm of PET images. Most of these PET images come from the same institution, and image smoothing technology was used in PET reconstruction. Although this technology can reduce image noise and improve the visual effect of images, it can make important features of the image (such as tumor boundaries) smooth and blurry, leading to an increase in bias and a decrease in contrast resolution, which may affect the extraction of key features by deep learning models. Furthermore, the features of CT images may be easier for models to recognize and extract because they are usually directly related to anatomical structures. PET images may have more abstract features due to the use of radioactive tracers, as they are related to biochemical processes, which may require more complex models to understand and predict. In summary, CT images typically have better data quality, higher spatial resolution, and clearer identifiable features. Therefore, our research results indicated that deep learning models based on CT images have the highest predictive performance. The combined efficacy of PET and CT is comparable to CT, possibly due to the current approach to PET and CT data fusion; we employed an early fusion strategy, which may have contributed to the suboptimal fusion performance. In future work, we plan to explore various fusion strategies, such as mid-level and late fusion and conduct comparative studies on these approaches to optimize the combined performance of PET and CT modalities.

Our research applies Grad-CAM to explain the process of model prediction as Grad-CAM provides clinicians with an intuitive way to understand the prediction process of deep learning models, helping them identify and understand the biological or anatomical features behind model predictions and thus better utilize these models to assist clinical decision-making in practice. However, the application of AI decision-making in clinical processes still faces some challenges. We need to find a balance between ethics and clinical practice, ensure patient consent, clarify responsibility attribution, and reduce the risk of misclassification while effectively integrating it into clinical workflows.

Our study has some limitations. Firstly, the exclusion of most patients resulted from undetected *EGFR* gene status, which inevitably introduces a selection bias. Secondly, owing to the irregular shape of some tumors, the ROI delineation process is difficult and time-consuming. Finally, we manually segmented the tumor, although deep learning offers the advantage of automatic feature extraction of the tumor without segmenting the tumor. In the future, we would like to compare the performance of deep learning models constructed with manual tumor delineation to those constructed without manual tumor delineation. Finally, our study mixed data from two institutions together for model construction and internal testing, lacking external data validation. In the future, we will collect more data from other institutions for external validation to demonstrate the robustness and generalizability of the model.

In our work, optimized deep learning–based algorithm was trained to predict the *EGFR* mutation status in patients with NSCLC using ^18^F-fluorodeoxyglucose (^18^F-FDG) PET/CT images from two institutions. The outcome suggests that our model approach is a workable method for predicting *EGFR* mutations. The model constructed on the basis of CT images has better performance than PET and similarly to PET + CT models. The deep learning approach outperformed traditional clinical methods. Deep learning methods have shown great potential in predicting *EGFR* mutation status. They not only can improve the accuracy of treatment decisions, reduce invasive procedures, optimize resource allocation, and promote the development of precision medicine. With the further maturity of technology, this method is expected to play a greater role in future clinical practice.

## Data Availability

The datasets used during the current study are available from the corresponding author on reasonable request. Requests to access these datasets should be directed to GZ, zhanggj15@lzu.edu.cn.
