# Corrigendum: Predicting epidermal growth factor receptor mutation status of lung adenocarcinoma based on PET/CT images using deep learning

**DOI:** 10.3389/fonc.2025.1564325

**Published:** 2025-02-12

**Authors:** Lele Huang, Weifang Kong, Yongjun Luo, Hongjun Xie, Jiangyan Liu, Xin Zhang, Guojin Zhang

**Affiliations:** ^1^ Department of Nuclear Medicine, The Second Hospital and Clinical Medical School, Lanzhou University, Lanzhou, China; ^2^ Key Laboratory of Medical Imaging of Gansu Province, Lanzhou, China; ^3^ Gansu International Scientific and Technological Cooperation Base of Medical Imaging Artificial Intelligence, Lanzhou, China; ^4^ Department of Radiology, Sichuan Provincial People’s Hospital, University of Electronic Science and Technology of China, Chengdu, China; ^5^ Department of Nuclear Medicine, Sichuan Provincial People’s Hospital, University of Electronic Science and Technology of China, Chengdu, China; ^6^ Department of Pharmaceuticals Diagnosis, GE Healthcare, Beijing, China

**Keywords:** ^18^F-FDG PET/CT, lung adenocarcinoma, EGFR, mutation, deep learning

In the published article “Nioche C, Orlhac F, Boughdad S, Reuzé S, Goya-Outi J, Robert C, et al. LIFEx: a freeware for radiomic feature calculation in multimodality imaging to accelerate advances in the characterization of tumor heterogeneity. Cancer Res.(2018)15;78(16):4786-89. doi: 10.1158/0008-5472.CAN-18-0125.” was not cited in the article. The citation has now been inserted in Section 2.6, *2.6 Data pre-processing* and should read:

“In the context of image preprocessing for CT data, the original images are first adjusted to a window width and level setting of −500 and 1,500, respectively. Following this adjustment, the data undergo a 1 × 1 × 1 resampling process. Subsequently, the CT images are cropped on the basis of the maximal edge range of the 3D ROI. For each case, eight central images from the lesion area are retained as input data (the eight central images of the lesion refer to the eightlayer coronal images of the central slice of the 3D lesion). Similarly, in the case of PET images, after completing the SUV conversion using LIFEx software (version v6.20) (22), the images are cropped on the basis of the maximal edge of the 3D ROI. Again, eight central images from the lesion are preserved as input data for each case. If the number of lesion slices is less than eight, then all slices of data are kept as input. The images are then resized to 299 × 299 pixels to satisfy the input requirements of the CNN model. The data are randomly stratified into training and validation sets in a 7:3 ratio.”

The authors apologize for this error and state that this does not change the scientific conclusions of the article in any way. The original article has been updated.

